# Does Natural Mentoring Matter? A Multilevel Meta‐analysis on the Association Between Natural Mentoring and Youth Outcomes

**DOI:** 10.1002/ajcp.12248

**Published:** 2018-04-25

**Authors:** L. Van Dam, D. Smit, B. Wildschut, S.J.T. Branje, J.E. Rhodes, M. Assink, G.J.J.M. Stams

**Affiliations:** ^1^ Spirit Youth Care Amsterdam University of Amsterdam Amsterdam The Netherlands; ^2^ Department of Child Development and Education University of Amsterdam Amsterdam The Netherlands; ^3^ Utrecht University Utrecht The Netherlands; ^4^ University of Massachusetts Boston Boston MA USA

**Keywords:** Natural mentoring, (Positive) youth outcomes, Adolescence, Community mental health, Prevention, Meta‐analysis

## Abstract

In this meta‐analytic review, we examined the relation between natural mentoring and youth outcomes in four domains: academic and vocational functioning, social‐emotional development, physical health, and psychosocial problems. Natural mentoring relationships are thought to foster positive youth development and buffer against the risks associated with the tumultuous years of adolescence. Two separate meta‐analyses were conducted on the presence of a natural mentor and the quality of the natural mentoring relationship, including thirty studies from 1992 to present. The findings indicated that the presence of a natural mentor was significantly associated with positive youth outcomes (*r = *.106). A larger effect size was found for the quality of the natural mentoring relationship in terms of relatedness, social support, and autonomy support (*r = *.208). The largest effect sizes were found for social‐emotional development and academic and vocational functioning. Risk‐status (e.g., teenage mothers, homeless youth, youth in foster care, and youth of alcoholic parents) did not moderate the relation between presence and quality of natural mentoring relationships and youth outcomes, which may indicate that natural mentors are generally beneficial for all youth regardless of risk‐status. Implications for theory and practice concerning the quality of the natural mentoring relationship are discussed.

## Highlights

- The presence of a natural mentor is associated with positive youth outcomes.
- A larger impact was found for quality of the natural mentoring relationship.
- Social‐emotional & academic and vocational functioning profited the most.
- On presence and relationship quality, risk‐status did not moderate youth outcomes.

## Introduction

In addition to parents, caring adults can play a vital role in the educational, behavioral, and emotional development of children and adolescents (Bowers, Johnson, Warren, Tirrell, & Lerner, 2015; Kesselring, De Winter, Van Yperen, & Lecluijze, 2016). Relationships with extended family members, teachers, coaches, and other adults increase in importance during adolescence, as adolescents are biologically, emotionally, and developmentally wired for engagement beyond their families, and increasingly gain psychological and behavioral autonomy from their parents (Bowers et al., 2014; Fruiht & Wray‐Lake, 2013; Patton et al., 2016). Connections between youth and caring non‐parent adults can develop into natural mentoring relationships that foster positive youth development and buffer against the risks associated with the tumultuous years of adolescence (Bowers et al., 2015).

Despite their ubiquity and the considerable progress in research on natural mentoring relationships, meta‐analytic studies of youth mentoring have focused almost exclusively on the impact of formal mentoring relationships. Meta‐analyses only found small overall positive effects of formal mentors on the psychological, emotional, behavioral, and educational functioning of participating youth (DuBois, Portillo, Rhodes, Silverthorn, & Valentine, 2011; Jolliffe & Farrington, 2007; Tolan et al., 2013; Wheeler, DuBois, & Keller, 2010). Particularly since research on the effects of natural mentoring during adolescence is steadily growing, and results have not been consistent across outcomes or in some instances were even equivocal (DuBois & Silverthorn, 2005a; Rhodes, Contreras, & Mangelsdorf, 1994; Zimmerman, Bingenheimer, & Notaro, 2002), a meta‐analysis seems timely to integrate the current knowledge on natural mentoring and explain differences within and between studies. Two separate meta‐analyses were conducted, first on the presence of a natural mentor and subsequently on the quality of the natural mentoring relationship, to examine associations between mentoring and academic and vocational functioning, social‐emotional development, physical health, and psychosocial problems.

A mentoring relationship is generally characterized as a strong connection between an older or more experienced individual who provides guidance and support to a younger or less experienced mentee or protégé over time (Rhodes, 2002). This conceptualization of youth mentoring encompasses approaches that vary in structure and context, ranging from formal relationships—in which mentees and mentors are matched and monitored through a program that outlines specific expectations about the parameters of the relationship (e.g., frequency and duration of contact)—and informal or natural mentoring relationships that form organically between youth and older individuals within their existing social networks. Natural mentoring requires fewer resources, and is far more accessible to a broader range of youth than formal youth mentoring (an estimated 75% of youth have natural mentors vs. roughly 5% with formal mentors (Erickson, McDonald, & Elder, 2009; Raposa, Dietz, & Rhodes, 2017).

A natural mentor may be a non‐parent relative, neighbor, teacher, friend, or someone from a religious community, who is a confidant and advocate for the youth (Hurd & Zimmerman, 2010b; Schwartz, Rhodes, Spencer, & Grossman, 2013; Spencer, Tugenberg, Ocean, Schwartz, & Rhodes, 2016; Van Dam et al., 2017). Natural mentors may provide support that enhances youth's sense of belonging and mattering with significant others (Bowers et al., 2012; Erikson, 1968; Lerner, Von Eye, Lerner, & Lewin‐Bizan, 2009). This support can range from informational, such as giving advice about work or education, and emotional, such as providing comfort and support, to instrumental, such as help applying for jobs or coping with day‐to‐day stressors (Erickson et al., 2009; Van Dam et al., 2017). By being a companion to youth and providing reliable support, natural mentors may help to foster a range of positive developmental outcomes and resilience (Southwick, Morgan, Vythilingam, & Charney, 2007). Through social interactions with natural mentors, adolescents acquire and refine new cognitive skills, and become more receptive to adult instruction and perspectives (Radziszewska & Rogoff, 1991). During adolescence, when identity development becomes central, mentor guidance may help shift youths’ conceptions of both their current and future identities and help them develop ideas of what they might become or would like to become in the future (Darling, Hamilton, Toyokawa, & Matsuda, 2002; Erikson, 1968; Markus & Nurius, 1986).

Further, natural mentors can provide a protective resource for youth who have had a history of difficult or unsatisfactory relationships (Southwick et al., 2007). By modeling, caring, and providing emotional support, natural mentors can challenge negative views that some youth may hold of themselves and others, and let them experience that positive relationships with adults are possible. The natural mentoring relationship may become a ‘corrective’ experience for youth who have a history of unsatisfactory relationships with parents or other caregivers (Rhodes, 2005). For these youth, natural mentors may function as secondary attachment figures who satisfy their emotional and social needs (Bowlby, 1988; Erdem, DuBois, Larose, De Wit, & Lipman, 2016; Rhodes, Spencer, Keller, Liang, & Noam, 2006). In this way, natural mentors may counteract or neutralize the relational risk that many youth face (Zimmerman et al., 2002).

Of course, not all mentoring relationships are the same, and their influence can vary as a function of relationship quality, mentor, and mentee characteristics (Goldner & Mayseless, 2009; Grossman, Chan, Schwartz, & Rhodes, 2012; Grossman & Rhodes, 2002; Hurd & Sellers, 2013; Parra, DuBois, Neville, Pugh‐Lilly, & Povinelli, 2002; Rhodes et al., 2006). Although formal mentoring relationships share certain key features and constraints, the range and quality of natural mentoring relationships can vary widely. Key features of relationship quality are emotional closeness, frequency of contact, support, and relationship duration (Rhodes, 2002). Youth may take advice more easily from their natural mentor when they feel more supported or experience a close emotional bond (Hurd & Sellers, 2013). Also, greater frequency of contact and longer lasting relationships are thought to create opportunities for more involvement and closeness between the mentor and youth (Whitney, Hendricker, & Offutt, 2011). The frequency of contact and length of the relationship may be essential for positive changes (Hurd & Zimmerman, 2010b; Karcher, Nakkula, & Harris, 2005; Spencer et al., 2016). The amount of support natural mentors offer may foster trust, empathy, and respect (Eby et al., 2013). Positive changes in the lives of youth are often the result of a supportive bond between the youth and his or her natural mentor (Higley, Walker, Bishop, & Fritz, 2014; Spencer & Rhodes, 2005).

Mentor characteristics have also been shown to contribute to the quality of the natural mentoring relationship. When natural mentors are more familiar with the youth's cultural and personal background (e.g., same ethnicity, same gender), natural mentors may have a better understanding of the support needed, and may provide more appropriate guidance (Whitney et al., 2011). The mentors’ kinship status can be influential in this regard. Family (kin) members serve as mentors more often for younger adolescents, whereas mentoring relationships with non‐familial (non‐kin) mentors and mentors with a helping profession background (e.g., teacher, guidance counselor, therapist) often develop during the middle and secondary school years (Fruiht & Wray‐Lake, 2013). Although kin relationship ties tend to be more intensive, they may be less able to serve as ‘bridging’ social capital that can link youth to a wider range of educational and occupational opportunities (Raposa, Erikson, Hagler, & Rhodes, 2018). This is consistent with the developmental stage of adolescence, when youths build identities outside the family and autonomy from parents increases considerably (Bowers et al., 2014; Hurd, Stoddard, Bauermeister, & Zimmerman, 2014).

Youth characteristics may influence both the quality of the natural mentoring relationship and its overall effects (Zimmerman, Bingenheimer, & Behrendt, 2005). High levels of individual, family, or neighborhood risk can be disruptive to relationships, contributing to greater mentor or youth dissatisfaction within their relationship (Raposa, Rhodes, & Herrera, 2016). This, in turn, may attenuate the positive effects of the natural mentor. Moreover, because many risk factors are relatively stable across time and context, they may impede the development of close ties and other protective factors over time (Vanderbilt‐Adriance & Shaw, 2008). Nevertheless, a particularly caring and consistent adult has the potential to play a significant role in the life of a youth who is experiencing high levels of stress (Greeson & Bowen, 2008). In this way, natural mentors could modify the relation between risk and outcomes by lessening the effect of risk factors and enhancing the effects of existing protective factors.

Finally, although typically unexamined in the mentoring literature, major factors that have consistently shown to affect meta‐analytic results in other fields of research are study characteristics, such as the year and quality of the study (Cheung & Slavin, 2016). It is expected that the quality of older studies is lower than the quality of more recent studies, as statistical methods and methodological knowledge have increased in social science research over the last decades (Saha, Saint, & Christakis, 2003). In addition, published studies in higher rated journals may report larger effects than unpublished reports due to biases in publishing only the stronger associations and significant results (Cheung & Slavin, 2016).

This study examined the relation between natural mentoring and youth outcomes in various domains of adolescent functioning, accounting for both within and between study differences in effect sizes. Variables that have been shown to be significant moderators in previous studies of informal mentoring as well as potential moderating variables that were neglected in past studies were examined to test which relational, individual, and study factors moderate the association between natural mentoring relationships and youth outcomes. Particular attention is given to the role of mentoring relationship quality in shaping youth outcomes.

We first hypothesized that the presence of a natural mentor, compared to having no mentor, would be positively associated with youth outcomes. Since the literature on natural mentoring relationships does not suggest negative influences, the quantification of the positive effects of natural mentoring on youth outcomes is a key benefit of our meta‐analytic study. Second, we hypothesized that the quality of the natural mentoring relationship would be positively associated with favorable youth outcomes. Third, based on previous research, we hypothesized that the involvement of non‐familial mentors, particularly natural mentors with a helping profession background, would result in stronger associations with positive youth outcomes than involvement of kin mentors. Finally, we explored the extent to which mentoring may be moderated by youth risk status.

## Method

### Sample of Studies

In the current meta‐analytic review, all studies addressing the relation between natural mentoring relationships and youth outcomes published before October 2017 were included, expect for non‐English studies. To find articles published in scientific journals, books, and unpublished reports, we used the following databases: ERIC, PsychINFO, PubMed, Wiley Online Library, and Google Scholar. Wildcards were used similarly across all databases. The search string included two elements: a mentor element and an outcome element. For the mentor element, the following terms were used: “natural mentor*,” “informal mentor*,” “youth mentor*,” “important non‐parental adult*,” “naturally acquired mentoring relationship*,” “mentoring adolescen*,” “VIP,” or ”YIM.” Similar to previous meta‐analyses of youth mentoring (DuBois & Silverthorn, 2005b; DuBois et al., 2011; Eby, Allen, Evans, Ng, & DuBois, 2008; Eby et al., 2013), the following keywords were used among others for the outcome element: “youth outcom*,” “behavior outcom*,” “academic outcom*” “foster care,” “youth care,” “delinquency,” “internalizing problem*,” “externalizing problem*,” “psychopathology,” “social‐emotional,” and “work‐related outcom*.” In addition, reference lists of the usable articles were inspected to find additional relevant studies. Authors were contacted to retrieve relevant studies and missing study information as much as possible. Four eligible studies were excluded, as they could not be traced in any digital library and their authors were unresponsive.

Multiple inclusion criteria were formulated to select the studies for this meta‐analysis. First, youth outcomes had to be operationalized as academic and vocational functioning, social‐emotional development, physical health, or psychosocial problems. Second, the mean age of the sample had to be between age 13 and 24. Third, the natural mentor had to be a different person than the youth's parents or step‐parents and fulfill the role of an important person in the life of the youth. Fourth, the connection between the mentor and youth had to be an already existing relationship. Studies measuring effects of mentoring programs with natural mentors were excluded.

A common problem in performing a meta‐analysis is that studies may not have been published because of non‐significant or unfavorable findings, the so called “publication or file drawer bias” (Rosenthal, 1995). Therefore, it is possible that the studies included in the meta‐analysis are not an adequate representation of all previously conducted studies on this topic. To reduce the problem of publication bias in our results, unpublished studies were screened by searching the ResearchGate database and several authors were contacted and asked for unpublished studies. Finally, the full publication lists of well‐known authors in the field of natural mentoring (i.e., DuBois, Hurd, Rhodes, Zimmerman) were screened for additional studies that could not be found in the databases.

The three‐first authors conducted the screening and selection process. When in doubt, the last authors were consulted. Appendix A presents a flow chart of the search. The initial search resulted in 281 articles, which also contained reviews and qualitative studies. This was narrowed down to 33 articles by inspection of the title and abstract. By using the ancestry method on these 33 articles, 39 new articles were included. By thoroughly examining full texts of the 72 studies, 42 studies were excluded because they did not fit the inclusion criteria. A total of 30 studies (with 222 effect sizes) met the inclusion criteria. Table 1 provides an overview of the included studies and their characteristics. Included studies in the present review are marked with an asterisk in the reference list.

**Table 1:** Characteristics of included studies

Author (year)	*N*	Peer review	IF	Design	Informant	Continent	Type of outcome	Sex	Ethnic minority	Mean age	Sample type
Presence natural mentor											
DuBois and Silverthorn (2005a)	3187	Yes	4.14	Long	Self	USA	Mixed	B/G	32.60	21.40	General population
Hurd and Sellers (2013)	259	Yes	1.56	Cross	Self/Teacher	USA	Mixed	B/G	100	13.56	General population
Zimmerman et al. (2002)	770	Yes	2.07	Cross	Self	USA	Mixed	B/G	82.80	17.50	General population
Ahrens et al. (2010)	1714	Yes	1.22	Cross	Self	USA	Mixed	B/G	25	16.00	General population
Ahrens, DuBois, Richardson, Fan, and Lozano (2008)	310	Yes	5.47	Cross	Self	USA	Mixed	B/G	35	16.00	At risk population
Rhodes et al. (1992)	129	Yes	2.15	Cross	Self	USA	Psychosocial problems	Girls	100	18.07	At risk population
Rhodes et al. (1994)	54	Yes	2.15	Cross	Self	USA	Mixed	Girls	100	18.10	At risk population
Collins, Spencer, and Ward (2010)	96	Yes	0.38	Cross	Interviewer	USA	Mixed	B/G	47	19.00	At risk population
Dang, Conger, Breslau, and Miller (2014)	197	Yes	1.28	Cross	Self	USA	Mixed	B/G	75.6	18.00	At risk population
Erickson et al. (2009)	12,621	Yes	2.86	Long	Official registration	USA	Academic	B/G	35	21.72	General population
Hurd and Zimmerman (2010a)	615	Yes	2.15	Cross/Long	Self	USA	Psychosocial problems	B/G	100	17.51	General population
Hurd and Zimmerman (2010b)	93	Yes	2.48	Cross/Long	Self	USA	Psychosocial problems	Girls	100	17.66	At risk population
Hurd et al. (2014)	3334	Yes	2.05	Cross	Self	USA	Mixed	B/G	24.6	20.80	General population
Hurd, Varner, and Rowley (2013)	259	Yes	3.56	Cross	Self	USA	Socio‐emotional	B/G	100	13.56	General population
Sánchez, Esparza, and Cólon (2008)	140	Yes	0.80	Cross	Self/Official registration	USA	Academic	B/G	100	17.88	General population
Cavell, Meehan, Heffer, and Holladay (2002)	95	Yes	1.33	Cross	Self	USA	Psychosocial problems	B/G	17	18.70	At risk population
Kogan, Brody, and Chen (2011)	375	Yes	2.15	Long	Self/Composite	USA	Mixed	B/G	100	17.00	General population
Greeson, Usher, and Grinstein‐Weiss (2010)	7977	Yes	0.97	Long	Self	USA	Vocational	B/G	20	21.28	General population
Hagler, Raposa, and Rhodes (2017)	193	–	–	Long	Self	USA	Mixed	B/G	43.5	11.20	General population
McDonald and Lambert (2014)	16,386	Yes	2.15	Long	Self	USA	Vocational	B/G	–	22.50	General population
Linnehan (2003)	47	Yes	2.76	Long	Self	USA	Mixed	B/G	79	17.45	General population
McDonald, Erickson, Johnson, and Elder (2007)	5740	Yes	1.77	Long	Self	USA	Vocational	B/G	35	‐	General population
Erickson and Phillips (2012)	8379	Yes	1.23	Long	Self	USA	Academic	B/G	47	15.36	General population
Munson and McMillen (2009)	339	Yes	0.97	Long	Self	USA	Mixed	B/G	55	19.04	At risk population
Relationship quality											
Hurd et al. (2013)	259	Yes	3.56	Cross	Self	USA	Socio‐emotional	B/G	100	13.56	General population
Chang, Greenberger, Chen, Heckhausen, and Farruggia (2010)	754	Yes	2.48	Long	Self	USA	Mixed	B/G	77	17.50	General population
Schwartz, Chan et al. (2013)	1860	Yes	1.97	Cross	Self	USA	Socio‐emotional	B/G	44.4	15.00	General population
Sánchez et al. (2008)	140	Yes	0.80	Cross	Self/Official registration	USA	Academic	B/G	100	17.88	At risk population
Kogan et al. (2011)	116	Yes	1.79	Cross	Self/Official registration	USA	Mixed	B/G	100	19.50	At risk population
Bowers et al. (2012)	710	Yes	1.97	Long	Self	USA	Socio‐emotional	Boys	21.1	15.77	General population
Black, Grenard, Sussman, and Rohrbach (2010)	3320	Yes	1.67	Long	Self	USA	Psychosocial problems	B/G	59	15.30	General population
Klaw and Rhodes (1995)	204	Yes	2.40	Cross	Self	USA	Mixed	Girls	100	15.90	At risk population

*N* = number of participants; peer review = published in peer reviewed article (Yes/No); IF = impact factor of journal; design = cross‐sectional (Cross) or longitudinal (Long); informant = self‐report (Self), interviewer‐report (Interviewer) or teacher‐report (Teacher); type of outcome = internalizing, conduct problems, overall psychopathology, substance use (Psychosocial problems), social confidence and/or confidence (Social‐emotional), Academic, Vocational or two or more different outcome domains (Mixed); sex = only girls (Girls), only boys (Boys) or boys and girls (B/G); Ethnic minority = proportion of non‐Caucasian. The study of Hagler, Raposa, & Rhodes is unpublished.

### Coding Studies and Potential Moderators

Two separate meta‐analyses were conducted to assess the overall relation between the role of a natural mentor and youth outcomes. The first meta‐analysis focused on the presence or absence of a natural mentor, in which most studies could be included. Since some studies focused on the quality and availability of a natural mentor (e.g., Likert‐scale of availability, less or more connected mentor), instead of the presence or absence, the second meta‐analysis was conducted to assess the relation between the quality of the natural mentoring relationship and youth outcomes.

The three‐first authors of this article coded the included studies according to the suggestions of Lipsey and Wilson (2001). The outcome variable in the meta‐analytic review was youth outcomes in several life domains. The predictor variable for the first meta‐analysis was the presence of a natural mentor, where the quality of the natural mentoring relationship was the predictor variable for the second meta‐analysis. Relationship quality was considered to consist of three dimensions: relatedness (e.g., closeness and trust between youngster and mentor), social support (e.g., degree of emotional, instrumental and cognitive support), and autonomy support (e.g., helping the youth to believe in his ability to achieve intended results through listening, modeling, and building confidence in the capabilities and efforts of the mentee). Five studies (*#ES = *36) were double coded by two of the first authors. It is common to calculate the inter‐rater agreement, which proved to be good with 94% agreement between the two coders on all outcome domains and moderator variables.

Each study was coded on multiple characteristics. The characteristics could be divided into five major categories: assessment of outcomes (type of domain); relationship characteristics (length of relationship, type of support, frequency of contact); mentor characteristics (type of mentor, ethnicity matched, gender matched); participant characteristics (percentage ethnic minority, percentage male, age, risk status); study characteristics (publication year, impact factor, published/unpublished, study design, type of reporter, type of measure, reliability, uni‐/multivariate).

Similar to other published meta‐analyses and studies on youth mentoring, outcome variables that were conceptually similar were combined (DuBois & Silverthorn, 2005b; DuBois et al., 2011; Eby et al., 2008, 2013). This was necessary to draw general conclusions about the relation between natural mentoring and youth outcomes. Table 2 lists the four broad categories of outcomes. For each category, the specific outcomes were examined and operationalized.

**Table 2:** Operationalizing of outcome domains, including examples of assessed variables in each domain

Domain	
Academic and vocational	High school completion, school attendance, academic engagement, higher grades, absences, school importance, school belonging, economic benefits, fulltime employment, discontinuous employment
Social‐emotional	Social skills, prosocial behavior, negative life events, self‐regulation, perceived social support, care, character, connection, life satisfaction, well‐being, self‐esteem
Physical health	General health, physical activity, birth control, condom use, Body Mass Index above 25, Sexually Transmitted Disease Diagnosis
Psychosocial problems	Depression, anxiety, suicidal ideation, psychosomatic symptoms, mental health, Sexual risk behavior, delinquency, problem behavior, aggression, rule breaking, Global severity, SCL‐90‐R, substance use

Several relationship characteristics were coded: length of the relationship in years (continuous variable), the percentage of informational, instrumental and/or emotional support youths receive from their natural mentor (continuous variables), and the frequency of contact (daily or weekly). Mentor characteristics, such as type of mentor, were coded into three categories: kin (e.g., grandmother, grandfather, aunt, uncle, older sibling), non‐kin (e.g., sport coach, employer, sport coach, co‐worker, neighbor, friend, friend's parents), and mentors with a helping profession background (e.g., teacher, guidance counselor, minister/priest/rabbi, religious leader, doctor/therapist). Also, the percentage of youth and mentors with the same gender and/or ethnicity was coded (continuous variable).

The following participant characteristics were coded: proportion of youth with a minority background (non‐white) in the sample (continuous variable), proportion of males (continuous variable), and the mean age. The capacity and willingness of youths to forge close connections with natural mentors may vary as a function of their age. At‐risk status was coded, as effects of natural mentors may differ for at‐risk youths and normally developing youths (DuBois & Silverthorn, 2005a; Werner, 1993). Youths were coded at‐risk when it was (explicitly) stated that the used sample was an at‐risk population. Examples of at risk populations were: American adolescent mothers, young Latino mothers, homeless youth, youth in foster care, youth of alcoholic parents, and pregnant and parenting teenagers.

At last, the following study characteristics were examined as moderators in the meta‐analysis. First, the year of publication (continuous variable) was coded. Second, the impact factor of the journal (the average across years) in which the study was published (continuous variable) was coded, because the impact factor is a first indication of study quality (Saha et al., 2003). Study design was coded (cross‐sectional vs. longitudinal design), as cross‐sectional studies measure the relation between natural mentoring and youth outcomes at one point in time, and longitudinal studies can take the developmental aspect of the relation between natural mentoring and youth outcomes into account.

### Calculation of Effect Sizes and Analyses

Effect sizes were transformed into the correlation coefficient *r*. A positive correlation indicated that the presence of a natural mentor or a higher quality of the natural mentoring relationship is associated with positive youth outcomes, whereas a negative correlation means that the presence of a natural mentor or a lower quality of the natural mentoring relationship is negatively associated with youth outcomes.

Effect sizes were calculated using the calculator of Wilson (2013) and formulas from Lipsey and Wilson (2001). If an article only mentioned that the relation was not significant, an effect size was coded as zero (Lipsey & Wilson, 2001). Continuous variables were centered around the mean, and categorical variables were recoded into dummy variables. Correlation coefficients *r* were recoded into Fisher *z*‐values (Lipsey & Wilson, 2001). After the analyses, the Fisher *z*‐values were transformed back into correlation coefficients for interpretation and reporting purposes. Standard errors and sampling variances of the effect sizes were estimated using formulas by Lipsey and Wilson (2001).

By including multiple effect sizes per study, the assumption of independent effect sizes that underlie classical meta‐analytic strategies was violated (Hox, Moerbeek, & Van De Schoot, 2010; Lipsey & Wilson, 2001). To deal with this interdependency of effect sizes and the differences in sample size, we applied a multilevel random effects model (Houben, Van Den Noortgate, & Kuppens, 2015; Van Den Bussche, Van Den Noortgate, & Reynvoet, 2009; Viechtbauer, 2010; Viechtbauer, 2010). This model is often used for multilevel meta‐analyses, and is generally considered superior to the fixed‐effects approaches used in traditional meta‐analyses (Van Den Noortgate & Onghena, 2003). A multilevel approach has the advantage of accounting for the hierarchical structure of the data by nesting effect sizes within studies. In this way, multiple effect sizes can be extracted from each included primary study, so that all information in the studies can be preserved and maximum statistical power is achieved (Assink & Wibbelink, 2016). A three‐level random effects model was used to account for three levels of variance, including the sampling variance of each effect size (level 1), the variance between effect sizes extracted from the same study (level 2), and the variance between the studies (level 3). The meta‐analyses were conducted in R (version 3.4.0) with the metafor‐package, using the syntax from Assink and Wibbelink (2016).

To estimate the model parameters, the restricted maximum likelihood estimate (REML) was applied (Van Den Noortgate & Onghena, 2003). The t‐distribution was used for testing individual regression coefficients of the meta‐analytic models and for calculating the corresponding confidence intervals (Knapp & Hartung, 2003). The Knapp and Hartung method (2003) has the advantage that it reduces Type I‐errors (Assink & Wibbelink, 2016). When models were extended with categorical moderators consisting of three or more categories, the omnibus test of the null hypothesis that all group mean effect sizes are equal followed an F‐distribution. Likelihood‐ratio tests were used to compare the deviance scores of the full model to the deviance of models excluding level 2 or level 3 variance parameters, making it possible to determine whether significant variance is present at the two levels (Assink & Wibbelink, 2016). In case there was significant variance on either of these two levels, the distribution of effect sizes was considered to be heterogeneous. This indicates that the effect sizes could not be treated as estimates of a common effect size, and univariate analyses for each moderator analyses were performed. To prevent the risk of chance capitalization, we chose to only examine a small set of relevant moderators, which are supported by literature.

Although several efforts were made to reduce publication bias in the search strategy, this could not guarantee the absence of publication bias or other forms of bias in the results. To assess the influence of publication bias, a funnel plot asymmetry according to Egger's method was tested first (Egger, Smith, Schneider, & Minder, 1997). A funnel plot is a scatter plot of the effect sizes against the effect sizes’ precision (the inverse of the standard error). In case of publication bias, a gap in the effect size distribution would be present, showing an asymmetrical funnel plot and a significant Egger's test. Second, a trim and fill procedure was performed, by drawing a trim and fill plot in MIX 2.0 (Bax, 2011; Duval & Tweedie, 2000). The trim and fill procedure corrects for funnel plot asymmetry by imputing estimated missing effect sizes that are calculated on the basis of existing effect sizes. If the trim and fill plot showed missing effect sizes, estimated effect sizes of missing studies were imputed, and the multilevel meta‐analyses were rerun in R, as this shows the influence of the estimated missing data on the overall effect of the meta‐analyses. Finally, the skewness of the effect size distribution was calculated in SPSS, because if publication bias is present, a skew distribution of the effect sizes would be expected (Begg & Mazumdar, 1994).

## Results

### Overall Relation Between The Presence of a Natural Mentor and Youth Outcomes

The meta‐analysis on the relation between the presence of a natural mentor and youth outcomes contains 24 independent studies (*k)*, reporting on 166 effect sizes (*#ES*), and a total sample of *N = *63,327 participants. A small, significant relation was found between the presence of a natural mentor and youth outcomes (*r *=* *.106, *p *<* *0.001; see Table 3). This indicates that the presence of a natural mentor was modestly, but significantly associated with more positive youth outcomes.

**Table 3:** Overall relation between the presence of a natural mentor on youth outcomes

Outcome	*k*	*#ES*	Mean *r*	95% CI	*p*	$\sigma_{\mathrm{level}\;2}^{2}$	$\sigma_{\mathrm{level}\;3}^{2}$	% Var. Level 1	% Var. Level 2	% Var. Level 3
Youth‐ outcomes	24	166	.106	0.076; 0.137	<.001***	0.008***	0.003***	2.94	69.33	27.73

Youth outcomes = academic and vocational, social‐emotional, physical health, psychosocial problems; *k =* number of studies; *#ES = *number of effect sizes; mean *r = *mean effect size (*r*); CI* = *confidence interval; $\sigma_{\mathrm{level}\;2}^{2}$ *= *variance between effect sizes extracted from the same study; $\sigma_{\mathrm{level}\;3}^{2}$ *= *variance between studies; % Var* = *percentage of variance distributed.

***p <* *.001

When checking for publication bias, Egger's test was not significant (*t = −*0.288, *p *<* *.774), indicating that there was no funnel plot asymmetry (Egger et al., 1997). Next, a trim and fill procedure was conducted. Appendix B shows 20 missing effect sizes on the right side of the funnel plot. When taking these 20 missing effect sizes into consideration, the overall effect was *r *=* *.148 instead of *r *=* *.106. These results suggest a small underestimation of the true effect.

The results of the likelihood‐ratio tests showed that there was significant variance between effect sizes from the same study χ^2^(1)* = *3,008.138, *p *<* *.001, and that there was significant variance between studies (i.e., level 3 variance), χ^2^(1)* = *19.796, *p *<* *.001. Since the variances at level two and three were significant, it was concluded that there was heterogeneity among the effect sizes that may be explained by characteristics of studies, samples, and natural mentoring relationships. Therefore, moderator analyses were conducted.

### Moderator Analyses on the Relation Between the Presence of a Natural Mentor and Youth Outcomes

Table 4 presents the results of the moderator analyses on the relation between the presence of a natural mentor and youth outcomes. Relationship characteristics did not have a moderating effect. For type of mentor, only the percentage of mentors with a helping profession background significantly moderated the relation between the presence of a natural mentor and youth outcomes. The risk‐status (general or at‐risk population) did not moderate the relation between the presence of a natural mentor and youth outcomes, indicating that the effect of the presence of a natural mentor did not differ for risk‐status. Last, none of the participant‐ or study characteristics moderated the relation between the presence of a natural mentor and youth outcomes.

**Table 4:** Moderators of the relation between the presence of a natural mentor and youth outcomes

Moderator variable	*k*	*#ES*	*B*_0_/mean *r*	*t*_0_	*B*_1_	*t*_1_	*F*(*df*_1_, *df*_2_)
Assessment of outcomes							
Domain							
Academic and vocational (RC)	15	52	.122	6.800***			*F*(3, 162)* = *2.642†
Social‐emotional	13	32	.126	5.424***	.004	0.157	
Physical health	4	14	.118	3.773***	−.004	−0.115	
Psychosocial problems	14	68	.069	3.552***	−.053	−2.446*	
Relationship characteristics							
Length relationship	8	78	.090	3.371**	.007	0.881	*F*(1, 76)* = *0.776
Informational support	8	53	.106	3.510***	.003	1.695†	*F*(1, 51)* = *2.874†
Instrumental support	6	48	.096	2.403*	−.000	−0.313	*F*(1, 46) = 0.098
Emotional support	8	55	.100	3.177**	−.000	−0.103	*F*(1, 53) = 0.0105
Amount of contact							
Predominantly daily (RC)	3	10	.135	3.219**			*F*(1, 60) = 2.151
Predominantly weekly	6	52	.065	2.544*	−.069	−1.467	
Mentor characteristics							
Percentage kin	17	122	.096	6.141***	−.000	−0.292	*F*(1, 120) = 0.085
Percentage non‐kin	14	102	.092	5.225***	−.000	0.014	*F*(1, 100) = 0.000
Percentage professional	14	101	.107	5.780***	.002	3.669***	*F*(1, 99) = 13.462***
Ethnicity matched	5	21	.060	1.033	.009	1.478	*F*(1, 19) = 2.184
Gender matched	5	30	.047	1.228	−.006	−1.231	*F*(1, 28) = 1.516
Participant characteristics							
% ethnic minority	22	159	.110	6.842***	.000	0.637	*F*(1, 157) = 0.405
% male sample	22	159	.110	7.184***	−.001	−1.200	*F*(1, 157) = 1.439
Age	22	165	.106	6.651***	−.003	−0.556	*F*(1, 163) = 0.309
Sample type							
General population (RC)	16	120	.091	5.323***			*F*(1, 164) = 2.637
At risk population	8	46	.144	5.212***	.053	1.624	
Study characteristics							
Publication year	22	163	.109	6.751***	−.000	−0.270	*F*(1, 161) = 0.073
Impact factor	22	163	.109	6.732***	.006	0.448	*F*(1, 161) = 0.201
Study design							
Cross‐sectional (RC)	13	99	.096	4.646***			*F*(1, 164) = 0.411
Longitudinal	11	67	.117	5.148***	.019	0.641	
Type of reporter							
Self‐report (RC)	21	149	.103	6.440***			*F*(2, 163) = 0.418
Other report/Teacher‐report	3	6	.110	1.822†	.001	0.011	
Official registration	2	11	.140	3.101**	.054	0.914	
Type of measure							
Single item (RC)	13	58	.097	4.523***			*F*(3, 148) = 0.451
Multiple items	5	16	.075	1.682	−.022	−0.657	
Scale	18	74	.111	5.675***	.013	0.574	
Index	2	4	.093	1.648	−.005	−0.080	
Reliability	16	68	.102	6.557***	−.067	−0.496	*F*(1, 66) = 0.246
Uni‐/multivariate							
Univariate (RC)	17	133	.105	6.028***			*F*(1, 164) = 0.031
Multivariate	7	33	.110	4.211***	.005	0.175	

*IV and DV characteristics* = independent variable (IV) and/or dependent variable (DV); *k *= number of independent studies; *#ES* = number of effect sizes; *B*_0_/mean *r = *intercept/mean effect size (*r*); *t*_0 _= difference in mean *r* with zero; *B*_1 _= estimated regression coefficient; *t*_1 _= difference in mean *r* with reference category; *F*(*df*_1_, *df*_2_) = omnibus test; (RC) = reference category.

^†^ *p *<* *.10; *p <* *.05; **p <* *.01; ***p <* *.001

### Overall Relation Between the Quality of the Natural Mentoring Relationship and Youth Outcomes

The meta‐analysis on the relation between the quality of the natural mentoring relationship and youth outcomes contains eight independent studies (*k)*, reporting on 56 effect sizes (*#ES*), and a total sample of *N = *7,363 participants. A small‐to‐medium, significant relation was found between the quality of the natural mentoring relationship and youth outcomes (*r *=* *.208, *p *=* *.002; see Table 5). This indicates that the quality of the natural mentoring relationship was significantly associated with more positive youth outcomes.

**Table 5:** Overall relation between the quality of the natural mentoring relationship on youth outcomes

Outcome	*k*	*#ES*	Mean *r*	95% CI	*p*	$\sigma_{\mathrm{level}\;2}^{2}$	$\sigma_{\mathrm{level}\;3}^{2}$	% Var. Level 1	% Var. Level 2	% Var. Level 3
Youth‐ outcomes	8	56	.208	0.144; 0.272	<.001***	0.006***	0.006***	5.48	47.12	47.40

Youth outcomes = academic and vocational, social‐emotional, physical health, psychosocial problems; *k =* number of studies; *#ES *= number of effect sizes; mean *r = *mean effect size (*r*); CI = confidence interval; $\sigma_{\mathrm{level}\;2}^{2}$ = variance between effect sizes extracted from the same study; $\sigma_{\mathrm{level}\;3}^{2}$ = variance between studies; % Var = percentage of variance distributed.

*p <* *.05; **p <* *.01; ***p <* *.001

When checking for publication bias, Egger's test was not significant (*t = *1.010, *p *=* *.317), indicating that there was no funnel plot asymmetry (Egger et al., 1997). Next, a trim and fill procedure was conducted. This procedure yielded no missing effect sizes, this is shown in Appendix B.

The results of the likelihood‐ratio tests showed that there was significant variance between effect sizes from the same study (i.e., level 2 variance) χ^2^(1)* = *338.911, *p *<* *.001, and that there was significant variance between studies (i.e., level 3 variance), χ^2^(1)* = *9.456, *p *<* *.01. Since the variances at level two and three were significant, it was concluded that there was heterogeneity among the effect sizes that may be explained by characteristics of studies, samples, and natural mentoring relationships. Therefore, moderator analyses were conducted.

### Moderator Analyses on the Relation Between the Quality of the Natural Mentoring Relationship and Youth Outcomes

Table 6 presents results of the moderator analyses on the relation between the quality of the natural mentoring relationship and youth outcomes. Type of outcome domain (social‐emotional, academic and vocational, and psychosocial problems) significantly moderated the association between the quality of the natural mentoring relationship and youth outcomes. Positive social‐emotional development showed a significantly larger (i.e., medium) effect size than psychosocial problems (i.e., small effect), whereas academic/vocational outcomes showed a small‐to‐medium effect. Relationship quality aspects and length of the mentoring relationship did not have a moderating effect. Also, type of mentor and risk‐status did not have moderating effects. Unexpectedly, higher reliability of instruments assessing youth outcomes was associated with smaller effect sizes.

**Table 6:** Moderating variables of relation between the quality of the natural mentoring relationships and youth outcomes

Moderator variable	*k*	*#ES*	*B*_0_/mean *r*	*t*_0_	*B*_1_	*t*_1_	*F*(*df*_1_, *df*_2_)
Assessment of outcomes							
Domain							
Social emotional (RC)	5	25	.264	7.685****			*F*(2, 53) = 5.098***
Academic and vocational	5	12	.196	4.852****	−.068	−1.329	
Psychosocial problems	3	19	.101	2.466*	−.162	−3.111***	
Relationship quality							
Relatedness	3	11	.205	3.219***			*F*(2, 53) = 0.036
Social support	3	26	.204	3.324***	−.000	−0.004	
Autonomy support	2	19	.228	2.943***	.024	0.236	
Relationship characteristics							
Length relationship	2	9	.170	2.8544	−.010	−0.937	*F*(1, 7) = 0.878
Mentor characteristics							
Percentage kin	5	20	.216	3.584***	−.001	−0.389	*F*(1, 18) = 0.152
Percentage non‐kin	3	11	.213	1.848†	−.005	−0.547	*F*(1, 9) = 0.300
Percentage professional	4	28	.187	1.914†	−.001	−0.371	*F*(1, 26) = 0.137
Participant characteristics							
% ethnic minority	8	56	.209	5.846****	−.000	−0.001	*F*(1, 54) = 0.001
% male sample	7	54	.208	5.954****	−.001	−0.124	*F*(1, 54) = 0.015
Age	8	56	.204	6.057****	−.031	−1.787†	*F*(1, 54) = 3.192†
Sample type							
General population (RC)	5	42	.195	4.696****			*F*(1, 54) = 0.326
At risk population	3	14	.237	4.024****	.041	0.572	
Study characteristics							
Publication year	8	56	.206	6.058****	−.004	−0.635	*F*(1, 54) = 0.403
Impact factor	8	56	.208	5.833****	.005	0.115	*F*(1, 54) = 0.013
Study design							
Cross‐sectional (RC)	5	21	.256	6.849****			*F*(1, 54) = 3.798†
Longitudinal	3	35	.149	3.701****	−.107	−1.949†	
Type of reporter							
Self‐report (RC)	8	53	.262	3.328***			*F*(1, 54) = 0.559
Official registration	2	3	.205	6.264****	.057	0.747	
Type of measure							
Single item (RC)	4	16	.153	3.902****			*F*(2, 50) = 1.943
Multiple items	2	2	.271	3.270***	.118	1.321	
Scale	8	35	.213	6.870****	.061	1.802†	
Reliability	8	35	.202	6.647****	−.549	−2.378*	*F*(1, 33) = 5.655*
Uni/multivariate							
Univariate (RC)	7	46	.218	6.103****			*F*(1, 54) = 0.548
Multivariate	1	7	.144	1.545	−.074	−0.740	

*IV and DV Characteristics = *independent variable (IV) and/or dependent variable (DV); *k *= number of independent studies; *#ES *= number of effect sizes; *B*_0_/mean *r = *intercept/mean effect size (*r*); *t*_0 _= difference in mean *r* with zero; *B*_1 _= estimated regression coefficient; *t*_1 _= difference in mean *r* with reference category; *F*(*df*_1_, *df*_2_) = omnibus test; (RC) = reference category.

^†^ *p *<* *.10; *p <* *.05; **p <* *.01; ***p <* *.001

### Moderator Analysis Between the Presence of a Mentor and the Quality of the Natural Mentoring Relationship

Analysis on the difference between the overall effect sizes of the presence of a natural mentor and the quality of the natural mentoring relationship shows that the overall effect sizes of the meta‐analyses significantly differ at *p *<* *.01: *F*(1, 220)* = *9.6434, *p *=* *.002. Therefore, the presence of a natural mentor yields a significantly smaller effect size than quality of the mentoring relationship.

## Discussion

The current meta‐analytic study examined the relation between the presence of a natural mentor, quality of the natural mentoring relationship and positive youth outcomes in four domains: academic and vocational functioning, social‐emotional development, physical health, and psychosocial problems. On the basis of a review of 30 studies (with 222 effect sizes) from 1992 to present, we found small effect sizes for the presence of a natural mentor and quality of the natural mentoring relationship. Larger effect sizes were found for the presence of a mentor with a helping profession background. Small‐to‐medium effect sizes were found for the association between quality of the natural mentoring relationship and social‐emotional development, academic and vocational functioning, and psychosocial problems. Finally, risk‐status did not moderate the relation between the presence of a natural mentor or the quality of the natural mentoring relationship and youth outcomes.

The results highlight the importance of natural mentoring relationships in the lives of youth, indicating that the presence of a natural mentor is related to positive outcomes and that the quality of the natural mentoring relationship can increase those positive outcomes. The finding that the presence of a natural mentor is related to positive youth outcomes is in line with the conclusions from a systematic review of natural mentoring in foster care (Thompson, Greeson, & Brunsink, 2016). Since the current meta‐analysis included nationally representative samples as well as specific risk and minority samples, the findings are applicable to youth in general. Furthermore, the positive finding for the quality of the natural mentoring relationship is consistent with a meta‐analysis on mentoring relationships in general (i.e., where no distinction was made between formal and natural relationships), which showed that high‐quality relationships were associated with more support and improved youth outcomes (Eby et al., 2013). Developing high‐quality relationships requires spending time and getting to know each other. The more frequently the mentor and the youth interact, and the more satisfying the relationship is, the greater the opportunity for the mentor to provide the youth with experiences of social support (Hurd & Zimmerman, 2010b).

The meta‐analysis on the presence of a natural mentor and youth outcomes yielded somewhat larger effect sizes for mentors with a helping profession background (e.g., teacher, guidance counselor, minister/priest/rabbi, religious leader, doctor/therapist), which may reflect the particular salience of caring teachers or guidance counselors in educational and community settings (DuBois, Holloway, Valentine, & Cooper, 2002; DuBois et al., 2011; Erickson, Karcher, Davis, & Powell, 2002). The findings are in line with a study on natural mentoring characteristics (DuBois & Silverthorn, 2005b), showing the benefits of relationships with natural mentors outside the family by building new (bridging) forms of social capital (McDonald & Lambert, 2014; Raposa et al., 2017).

Risk‐status did not prove to be a significant moderator. The absence of a significant moderating effect may indicate that natural mentors are generally beneficial for all youth regardless of risk‐status. On the one hand, natural mentors may serve as complementary resources and promote resilience when youth have good familial relationships. On the other hand, natural mentors may serve as compensatory resources for youth from backgrounds of risk or for whom parents may not be fully engaged in their lives (Britner, Balcazar, Blechman, Blinn‐Pike, & Larose, 2006; DuBois et al., 2002; Erickson et al., 2009). Although, youth with a history of unavailable or inconsistent care may be less likely to turn to others in times of stress (Belsky & Cassidy, 1994), natural mentors who are trustworthy and consistent may help youth feel more confident and open to emotional support when facing stressful events or chronic adversity (Rhodes et al., 2006; Rutter, 1987).

The overall small to moderate effect sizes for the relation between presence (*r *=* *.106) and quality (*r *=* *.208) of natural mentoring and positive youth outcomes are on the higher range if compared to formal mentoring effect sizes that have been reported over the past 30 years. These effect sizes range between *r *=* *.03 for the association between formal mentoring and psychological stress and *r *=* *.15 for the association between formal mentoring and aggression (DuBois et al., 2011; Eby et al., 2008; Jolliffe & Farrington, 2007; Tolan et al., 2013; Wheeler et al., 2010). A recent multilevel analysis, testing significant moderators from previous studies, as well as those that may have been neglected in past studies, showed a statistically significant, but small effect of mentoring programs on a range of youth outcomes: psychological, social, cognitive, health, or school, *r *=* *.09 (Raposa, Erikson et al., 2018; Raposa, Stams et al., 2018).

Given that natural mentoring relationships show small to moderate associations with positive youth outcomes and are far more common and require less infrastructure and investment than formal mentoring relationships, it seems important to strengthen the “relational capacity” of the everyday settings of youth and foster opportunities for natural mentoring relationships to develop. This can be done by improving the ratios and training of adults in schools and other developmental settings. For example, efforts to strengthen teacher‐student relationships in schools have been shown to have a substantial and positive impact on students’ academic achievement (Cornelius‐White, 2007; Roorda, Koomen, Spilt, & Oort, 2011), behavior problems (Cornelius‐White, 2007; Lei, Cui, & Ming, 2016), and social‐emotional development (Ahnert, Harwardt‐Heinecke, Kappler, Eckstein‐Madry, & Milatz, 2012; Cornelius‐White, 2007; Jennings & Greenberg, 2009; McGrath & Van Bergen, 2015). Efforts to improve adult‐youth relational opportunities in extracurricular informal learning activities are recommended (Clarijs, 2008; European Commission, 2015), as are efforts that enhance natural mentoring relationships in health, juvenile justice, and other settings where youth and adults routinely interact (Spencer et al., 2016; Van Dam et al., 2017), where adults have the potential to facilitate a range of benefits. Unfortunately, such settings are often structured in ways that diminish the potential opportunities for caring adult‐youth relationships. They are unevenly funded and lack training, standards, or incentives for forging close adult–youth relationships.

The results of this meta‐analytic study must be viewed within the context of the limitations associated with the empirical studies on which these meta‐analyses were based and the meta‐analyses there self. First, as noted, a clear and well‐validated definition of what constitutes natural mentoring relationships is absent in the field of natural mentoring, which makes it difficult to include all available quantitative studies (Thompson et al., 2016). For example, in some studies natural mentors had to be at least 20 years; 5 years older than the youth; or known to the youth for at least 2 years (Ahrens, DuBois, Lozano, & Richardson, 2010; Hurd & Zimmerman, 2010b; Rhodes, Ebert, & Fischer, 1992). In this way, peers could not be included as natural mentors. In a study by Whitney et al. (2011), the effects of peer mentoring on self‐esteem were even larger when compared to adult mentors. Second, risk‐status could not unambiguously be defined, particularly since some studies sampled subgroups of the general youth population (e.g., pregnant and parenting teenagers, homeless youth, children of alcoholics). This limits the generalizability of the findings, and the possibility to further examine the effects of risk‐status in terms of environmental or individual risk. Consequently, the results regarding the relation between natural mentoring and risk‐status deserve careful interpretation. Third, the hypothesis regarding the type of mentor could not be fully tested, since there was only one study in the field of natural mentoring that tested the effects of various types of mentors (e.g., kin‐mentor vs. non‐kin mentor) on youth outcomes (DuBois & Silverthorn, 2005b), and only four studies that tested a specific type of mentor compared to no mentor. Overall, results should be interpreted in light of self‐selection bias: those youth who recruit natural mentors may be higher functioning.

There are also some methodological limitations of this study that deserve consideration. First, for the meta‐analysis on the presence of a natural mentor, 20 effect sizes were estimated to be missing on the right side of the funnel plot when examining possible publication bias (see Appendix B). The missing effect sizes resulted in an underestimation of the true effect size, and therefore should be interpreted as selection bias given that missing studies that result in a smaller overall mean effect size would indicate publication bias. Selection bias may be due to an overrepresentation of certain samples or groups (e.g., data from the Adolescent Health Study). Second, there are limitations with respect to the generalizability of the study findings. The total sample consisted of 24 studies for the meta‐analysis on presence of a natural mentor, and only eight studies for the meta‐analysis on the quality of the natural mentoring relationship, which should lead to careful interpretation of the overall findings. Notably, we did not conduct multivariate moderator analyses, examining the unique impact of significant moderators, due to missing values across moderators and some moderator analyses were based on a small number of effect sizes (i.e., less than three effect sizes or studies), which reduces statistical power to detect a moderator effect and restricted generalizability of research findings. Last, all studies were English and conducted in the United States, potentially limiting generalizability to other countries or continents.

Despite these limitations, this study yielded promising findings with implications for future research. First, research on natural mentoring outside the US will facilitate comparisons between countries. Future research should more thoroughly examine the effects of natural mentoring on youth outcomes against levels of individual and environment risk and protective factors, to be able to examine the type of influence natural mentors may have, ranging from promotive to protective, depending on the level of adversity and number of risk and protective factors youths have (Hurd & Sellers, 2013). For example, with normally developing youth, natural mentors may play a significant role in helping youths cope with difficulties, achieve goals, and navigate their identity. For youths at‐risk, the natural mentoring relationship has the potential to offset individual and contextual risks, with adolescents often attributing their capacity to thrive despite adversity to the support of a caring adult (Greeson & Bowen, 2008).

This meta‐analysis showed that relationship quality is an important predictor of youth outcomes, as it increases the benefits from the natural mentoring relationship. Nevertheless, a shared (agreed upon) definition of mentor–mentee relationship quality is still missing and would strengthen our understanding of its role. The operationalization of relationship quality in natural mentoring studies comes down to three dimensions (i.e., social support, autonomy support, and relatedness) that may have a link with self‐determination theory (Ryan & Deci, 2000) and/or attachment theory (Bowlby, 1969/1982). Self‐determination theory—with its focus on competence, autonomy, and relatedness as basic needs to achieve positive (youth) development (Ryan & Deci, 2000)—may provide a theoretical base to understand the development of relationship quality in natural mentoring and the working mechanisms through which mentor–mentee relationship quality can exert a positive impact on various youth outcomes. Moreover, as supportive presence of the mentor (as a secure base) and relatedness (as a secure haven) appear to be important relationship dimensions affecting positive youth development, the mentor–mentee relationship may also be studied from the perspective of attachment theory. It can be hypothesized from recent developments in attachment theory that a mentor who is both sensitive to the emotional needs of the mentee and mind minded—which is the ability to treat other individuals with a mind of their own (Meins, 1997)—may foster a secure internal working model of attachment in his or her mentee, that is, a positive view of oneself as lovable and worthwhile and others as available and caring (Zeegers, Colonessi, Stams, & Meins, 2018). Subsequently, these secure working models of attachment may result in positive youth outcomes in several domains of functioning (e.g., Groh et al., 2014) or buffer against psychosocial problems (Colonnesi et al., 2011; Fearon, Bakermans‐Kranenburg, Van IJzendoorn, Lapsley, & Roisman, 2010; Hoeve et al., 2012).

Likewise, additional mentor characteristics (e.g., psychological well‐being, deviant behavior, having a job employment, school completion) should be studied more systematically to determine which characteristics of the natural mentor could result in their relative contribution to successful (i.e., supportive or protective) relationships. Notably, when natural mentors are engaged in problem behavior (i.e., substance use, delinquency), youth are more likely to be negatively affected by such deviant behaviors, which may be ascribed to negative role‐modeling (Chen, Greenberger, Farruggia, Bush, & Dong, 2003; Sterrett, Jones, McKee, & Kincaid, 2011).

In sum, these meta‐analyses advance our understanding of the important role of natural mentoring in the lives of youth and the conditions under which they are more impactful. The effects of natural mentoring on youth outcomes were relatively modest, but when relationship quality was taken into account, the effects of mentoring were considerably larger, particularly for positive social‐emotional development. In some cases the effects of high‐quality mentoring relationships exceeded many that have been reported in meta‐analyses and reviews on the association between formal mentoring relationships and youth outcomes. Taken together, these findings highlight the importance of ensuring that all youth, not just those who have access to networks with high social capital, have access to caring teachers, employers, and other adults who can serve as role models and have the relationship skills to provide developmental opportunities. Along these lines, efforts that encourage and teach youth how to recruit natural mentors, and mobilize adults in more freely sharing their social resources, represent promising directions for community mental health intervention (Schwartz, Chan, Rhodes, & Scales, 2013; Schwartz, Rhodes et al., 2013; Van Dam et al., 2017). Taken together, these initial findings challenge us to further understand the working mechanisms of natural mentoring and provide hope about the capacity of natural mentors to improve and even transform young lives.

## Appendix A

*Note*: Appendix A presents a flow chart of the search, showing the initial search (281 articles) resulting in a total of 30 studies (with 222 effect sizes) meeting the inclusion criteria.

## Appendix B

*Note*: The “Funnel plot presence of the mentor” shows 20 missing effect sizes on the right side of the funnel plot, suggesting a small underestimation of the true effect.

*Note*: The “Funnel plot quality of mentoring” shows no missing effect sizes, suggesting no underestimation or overestimation of the true effect.
